# Beneficiaries’ perceptions and reported use of unconditional cash transfers intended to prevent acute malnutrition in children in poor rural communities in Burkina Faso: qualitative results from the MAM’Out randomized controlled trial

**DOI:** 10.1186/s12889-017-4453-y

**Published:** 2017-05-30

**Authors:** Audrey Tonguet-Papucci, Freddy Houngbe, Palamanga Lompo, Wambi Maurice Evariste Yameogo, Jean-François Huneau, Myriam Ait Aissa, Patrick Kolsteren

**Affiliations:** 1 Research and Analyses Department, Action Contre la Faim, 14/16 Boulevard Douaumont - CS 80060, 75854 Paris CEDEX 17, France; 2 Department of Food Safety and Food Quality, Ghent University, Campus Coupure, A2.081, Coupure Links 653, B 9000 Ghent, Belgium; 3 UMR Physiologie de la Nutrition et du Comportement Alimentaire, AgroParisTech, INRA, Université Paris-Saclay, 16 rue Claude Bernard, 75005 Paris, France; 4 Institut de Recherche en Sciences de la Santé, Ouagadougou, BP 7192 Burkina Faso; 5 Action Contre la Faim | ACF-Burkina Faso, Rue 13-22, P557 Zogona Zone du bois, 06, Ouagadougou 06, BP 10221 Burkina Faso

**Keywords:** Unconditional cash transfer, Acute malnutrition, Children, Women, Burkina Faso, Perceived changes

## Abstract

**Background:**

Acute malnutrition is a public health issue worldwide, and particularly in the Eastern region of Burkina Faso. Following a needs assessment, unconditional seasonal, multiannual cash transfers were implemented as a safety net to prevent childhood undernutrition. The objectives of this study were to explore the types of purchases made by beneficiaries of this cash transfer program and to understand the perceived effects of and changes induced by regular cash transfers in the daily lives of women, and at the household and community level.

**Methods:**

The design of this study was a two-arm cluster randomized controlled trial. Qualitative data were collected each month during the cash transfer period for two years, leading to a total of more than 300 interviews and focus group discussions with various participants: beneficiary mothers, heads of households, mothers-in-law, co-wives, key members of the community, and participants of the control group.

**Results:**

The two main types of expenses reported were food and health care for the child and the whole family. The program was also associated with positive perceived changes at the household level, mainly related to gender equality and improvement of women’s status, and has promoted an increase in dignity and social integration of the poorest at the community level through cash sharing. Unexpected effects of this program included some women planning new pregnancies and some individuals not expecting the transfers to end.

**Conclusion:**

Although the transfers were unconditional, the cash was mainly used to improve the children’s and households’ food security and health, which correspond to two main underlying causes of undernutrition. Therefore, spending mainly in these areas can help to prevent undernutrition in children.

**Trial registration:**

ClinicalTrials.gov, identifier: NCT01866124, registered May 7, 2013.

**Electronic supplementary material:**

The online version of this article (doi:10.1186/s12889-017-4453-y) contains supplementary material, which is available to authorized users.

## Background

For several decades, humanitarian agencies have implemented food assistance programs for populations in need, as part of development and/or humanitarian responses. These programs can take various forms, from direct food distribution when no food is available in the affected area to, more recently, vouchers or cash transfers when markets are functional and food is available locally. These vouchers and transfers are often restricted to specific items or to food, and come with guidance on how to use the money. However, needs vary greatly between families, depending on their individual situations and the composition of their households. In this respect, unconditional and unrestricted cash transfers allow beneficiaries to spend the cash that they receive according to their own priorities and needs. This approach was chosen in the Eastern region of Burkina Faso, within the framework of the MAM’Out (Moderate Acute Malnutrition Out) research project [1].

With around 17 million inhabitants, Burkina Faso is one of the poorest countries in the world. On the human development index, it ranks 181 out of 187 nations [2]. The prevalence of wasting is high: at the country level, 10.2% of children under 5 years old were acutely malnourished (2006 WHO growth standard) in August/September 2011 (which corresponds to the end of the hungry season) [3], and this prevalence reached 17.3% in April 2012 in the Tapoa Province in the Eastern region of Burkina Faso [4], which makes the region a priority area in terms of tackling undernutrition. The main ethnic group in the Tapoa province is the Gourmantché, with Pulaar people represented to a lesser extent. Most of the Gourmantché people live in villages run by key male members of the community, namely the village head, supported by members of the village development council. A village is comprised of numerous concessions. A concession is a group of huts linked together by a fence forming a round courtyard where an extended patrilineal family lives, i.e. the “old” parents and their sons with their entire family (wives and children), and sometimes other family members [5]. Each concession contains several households, and each household has a head of household (usually the husband). The households are predominantly polygamous. The husband is usually the principal decision-maker for the household, although he remains under the influence of his father. The husband is in charge of providing millet to feed his wives and children, and is responsible for his wives’ actions. Each wife has a separate hut where she raises her children and keeps some of the food assigned to them. Two situations may occur. If each household within the concession has its own field and produces grains and vegetables, then each household is independent, and when the co-wives prepare meals, they have to give some to their parents-in-law. However, if all of the sons work in their father’s fields, the granary is shared and handled by the head of the concession. In this case, meals are taken together, and the wives from each household take turns preparing meals for the whole concession. Traditional meals are based on grains eaten along with a sauce. Rivalry and tensions between co-wives may arise, even if the husband supposedly has to treat them equally. The first wife assists her husband with ritual functions during ceremonies and supervises the sharing out of the cooking tasks. However, she does not have absolute authority over her co-wives [6]. The relationship between co-wives, as well as gender practices (the relationship between and roles and responsibilities of men and women) are thus crucial when implementing and monitoring a program within the Gourmantché community.

In 2012, the Burkinabe national government, supported by a group of technical and financial partners, launched a national policy for social protection, with an action plan that identified the improvement of social transfer mechanisms for the poorest and the most vulnerable, in order to ensure food security, as a priority [7]. In this context, the MAM’Out project tested the distribution of unconditional multiannual, seasonal cash transfers, which are intended to act as safety nets to conserve household resources, at an appropriate, predictable and guaranteed level. It was assumed that, during the hungry season, household expenditures would exceed income, leading to the implementation of damaging coping strategies that increase the risk of child malnutrition. Presumably, predictable cash transfers during this key period would prevent the implementation of these negative strategies, and could even support longer term investment in productive assets and/or positive practices.

Cash transfers have already been proven to be effective in reducing poverty, and have the potential to support livelihoods and promote food security [8]. However, how cash transfer programs affect the daily life of communities benefiting from these transfers is rarely explored in depth. In the MAM’Out study, our aim was to obtain a more comprehensive understanding of how cash transfers were perceived and used, and to identify any unexpected effects of these transfers. More specifically, we sought to describe the types of purchases made to prevent undernutrition in children and to understand the effects and changes induced by regular cash transfers in the daily lives of women, the household, and the community, with regard to preventing undernutrition in children. For this reason, the qualitative study nested within the MAM’Out trial focused mainly on the cash group, although data were also collected from women in the control group in order to receive their feedback on the program and any unanticipated effects. Given the asymmetry in data collection between the two arms of the study, and the objectives of the qualitative study, the control and intervention groups were not compared in this study.

## Methods

### Ethics statement

Qualitative data were collected between July 2013 and November 2014. The qualitative study was performed within the framework of a larger research study (the MAM’Out research project), which received ethical approval from two separate ethics committees. The study was approved in April 2013 by the Ethical Committee of the University Hospital of Ghent, and in May 2013 by the Burkinabe National Ethical Committee. The study was registered with ClinicalTrials.gov (trial number: NCT01866124) on May 7, 2013. Mothers of children included in the MAM’Out study provided written informed consent prior to the enrollment of their children in the program. The different participants involved in the qualitative data collection also provided oral consent.

### The MAM’Out study: A mixed methods approach

The aim of the MAM’Out research project was to evaluate a seasonal, multiannual cash transfer program that was implemented to provide a safety net for preventing acute malnutrition in children under 36 months of age in the Tapoa province (Eastern region of Burkina Faso) [1]. The program began in May 2013, and targeted economically vulnerable households (i.e. poor and very poor households, as defined by the Household Economy Approach [9]) with children younger than one year old at the time of inclusion. All households meeting both criteria and belonging to the selected villages were invited to participate in the project. In total, 1178 children were included in the study. Cash was distributed during two consecutive hunger gaps to mothers via mobile phones, in collaboration with a mobile phone company. After receiving a text message informing them that the cash was available, the mothers had to take their mobile phone and SIM card to the nearest cash point to withdraw the money within a week’s time. The transfers were unrestricted, so the beneficiaries could decide how to use them. Women were chosen as the primary recipients of the transfers, as they are the primary child caregivers. The design of the study was a two-arm cluster randomized intervention trial. The randomized units were rural villages in the Tapoa province. In sixteen villages that were included in the intervention group, mothers received 10,000 FCFA (around €15) every month from July 2013 to November 2013 and from July 2014 to November 2014 for each child included in the study. Sixteen other villages were included in the control group. All families were followed up for two years. The main outcomes were the cumulative incidence of acute malnutrition (or wasting) and the cost-effectiveness of the program. Clinical outcomes of the trial will be reported elsewhere.

Quantitative data were collected quarterly from June 2013 to September 2015, based on a conceptual framework of actions of cash transfers to prevent acute malnutrition in children. Qualitative data were also collected. This mixed methods approach will allow for a more comprehensive understanding of the results and will help assess the validity of the findings [10]. Comparing qualitative findings between the control and the intervention groups is beyond the scope of this article, so the results will focus on the data collected from the intervention group. The important aspects of the qualitative study are reported according to the COREQ checklist [11].

### Theoretical framework of action of cash transfers

**Fig. 1:**
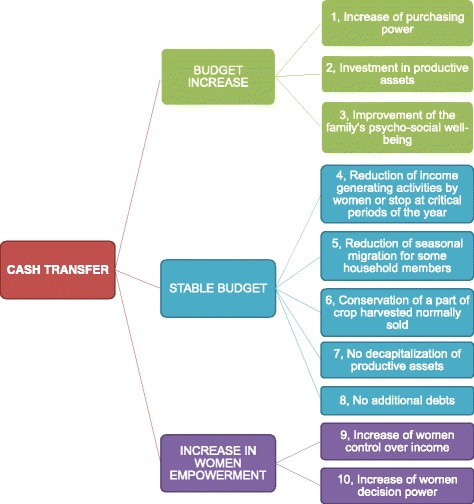
Simplified theoretical framework of action of cash transfers

While designing the study, a theoretical framework was constructed of how the cash transfers would work (Fig. 1), based primarily on existing literature and reports on the study area. A nutrition causal analysis conducted in the Tapoa province from November 2012 to December 2012 provided more detailed information about the pathways through which cash transfers can address acute malnutrition in the local context [12]. Three main hypotheses were generated. First, cash transfers can increase the household’s budget, leading to an increase in purchasing power, investment in productive assets, and improvement in the family’s psychosocial well-being. Secondly, cash transfers may help the household stay within its budget, preventing the family from taking out additional credit, selling productive assets, or migrating for work. Thirdly, cash transfers were hypothesized to increase female empowerment through the women’s control over the income and increased decision-making power.

This theoretical framework was used to analyze and organize the qualitative data collected. Individual interviews and focus group discussions offered an opportunity to evaluate this hypothesized model of the effects of cash transfers on preventing acute malnutrition in children. For this purpose, a semi-structured questionnaire administered in the local language was used to assess the experiences related to all possible cash pathways. Interview guides were designed based on the conceptual framework of how the transfers work, and were improved by adding new themes as they arose. More details on these questionnaires are provided later in the paper.

### Participants

**Fig. 2:**
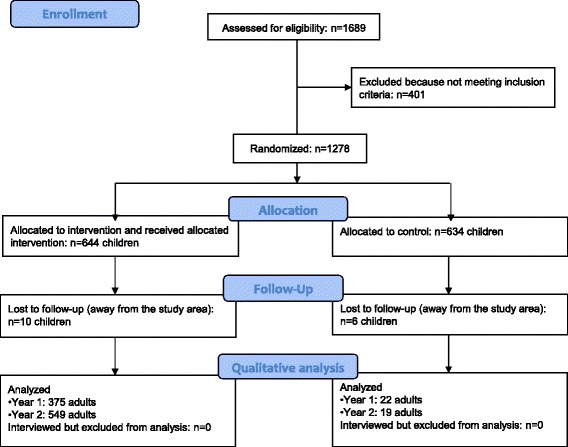
CONSORT flow diagram of the MAM’Out project qualitative data collection

In total, 375 people from the villages that received cash transfers participated in the qualitative data collection during the first year of the program and 22 people from the control group. The second year, 549 people from the villages that received cash transfers and 19 people from the control villages were involved (Fig. 2).

The choice of participants depended on the interviewers, who randomly selected families to interview. Lists of all the mothers from the different villages were obtained and completed with additional columns for husbands, co-wives, mother-in-law, etc. Participants were randomly selected by trained interviewers on a weekly basis. Once a participant was interviewed, his or her name was marked accordingly, and the lists were updated for reference for further randomization sessions. No individuals were interviewed twice in the same year. Seven to twelve participants were involved in each focus group discussion. Three groups of participants were included: 1/Direct beneficiaries of the cash transfers, i.e. mothers of the children who were followed up within the intervention group; 2/Indirect beneficiaries of the transfers: other households members benefiting from cash transfers (heads of household, co-wives, mothers-in-law) and key members of beneficiary villages (members of complaint committees and targeting committees, members of the village development councils); 3/Control group participants; more precisely, the mothers and heads of households of children who were followed up with as part of the study.

In total, 306 discussions were carried out, recorded and analyzed over the two-year program: 77 focus groups and 188 individual interviews from the intervention group, and 41 individual interviews from the control group. Table 1 summarizes the distribution of these discussions by category of interviewee, type of discussion and time of data collection. Most of the discussions occurred within the intervention group: 46% with indirect beneficiaries of the transfers and 41% with the cash recipients (the mothers). Only 13% of the discussions were held in the control villages.

**Table 1:** Number of participants in the focus groups and individual interviews conducted during the two-year project, classified by type of participant

	Type of participants	Intervention group		Control group	
		Year 1^a^	Year 2^a^	Year 1	Year 2
Focus group discussions (FGD)	Mothers	215 (25)	270 (25)		
	Heads of households	49 (6)	87 (9)		
	Co-wives	16 (2)	73 (7)		
	Mothers-in-law		26 (3)		
Individual interviews (II)	Mothers	32	43	22	17
	Heads of households	30	18		2
	Co-wives	2	6		
	Mothers-in-law		13		
	Key village members	31	13		
Total number of FGD and II		128	137	22	19

^a^The numbers in parentheses represent the number of focus groups held by type of participants and per year

The characteristics of the focus group discussions and individual interviews subsamples are similar to those of the MAM’Out population in term of socio-economic and matrimonial status, except for a slightly higher percentage of monogamous households and lower percentage of polygamous households participating in the focus group discussions (Table 2). On this basis, qualitative study subsamples can be considered fairly representative of the whole MAM’Out population.

**Table 2:** General characteristics of participants in the focus group discussions and individual interviews over the two years of project implementation compared to the global sample of the MAM’Out study at inclusion

	Participants in the individual interviews	Participants in the focus group discussions	MAM’Out population (at inclusion)
Matrimonial status of the head of household			
Monogamous, n (%)	98 (60,1)	506 (75,3)	758 (60,7)
Polygamous, n (%)	63 (38,7)	166 (24,7)	476 (38,1)
Widow, n (%)	2 (1,2)	0 (0)	11 (0,9)
Divorced, n (%)	0 (0)	0 (0)	4 (0,3)
Household socio-economic status (HEA criteria)			
Very poor, n (%)	46 (27,5)	155 (23,4)	322 (25,8)
Poor, n (%)	121 (72,5)	507 (76,6)	927 (74,1)
Medium, n(%)	0 (0)	0 (0)	1 (0,1)

The majority of participants belonged to the Gourmantché ethnic group, and only a few of the participants were Pulaar. When analyzing the results, no distinction was made between these two ethnicities, as very few data were obtained from Pulaar participants.

Systematic focus groups were held in all of the villages belonging to the intervention group. Sixteen villages received cash transfers, and focus groups were held in eight of these villages every other month, from July to November 2013 and July to November 2014, corresponding to the cash transfer periods. Hence, at least two focus groups were carried out each year in each village benefiting from the transfers. In parallel, individual interviews were conducted in the intervention and control villages to obtain a more detailed understanding of the cash utilization and any unexpected and/or negative aspects associated with these transfers.

### Focus groups and individual interviews

Qualitative data were collected monthly during the cash transfer periods via focus group discussions and individual interviews conducted by two interviewers (one male and one female) each year, who were employed full time by the study. All interviewers were Gourmantché from the Eastern region of Burkina Faso, had at least completed the final year of high school, and had at least one year of professional experience conducting focus groups. During the second year of the project, one interviewer had a master’s degree in sociology and had received a formal education in qualitative data collection. For both years of the study, the interviewers were trained for one week by skilled sociologists in qualitative data collection methods, and particularly in techniques used to conduct focus group discussions and individual interviews. Questionnaires were pilot-tested, and role-playing exercises were organized in order to identify potential difficulties and ways in which the interviewers could improve. The main difficulties that were encountered were the interviewers’ ability to follow up on interviewees’ answers and keep discussions going, and ensuring that all participants, particularly those who were shy about talking, were heard “equally”. The interviewers improved their skills in these areas primarily through additional practice. During the second year of the study, a sociologist supervised the process to improve the quality of data collection in the field.

Focus groups and individual interviews were conducted based on discussion guides that were designed prior to the beginning of the qualitative data collection and were based on the theoretical model of action of cash transfers. These discussion guides were first written in French, then translated into the local language (Gourmanchema) to ensure that they were understood by the interviewers (Additional file 1). Among the themes addressed during each focus group and individual interview were the definition of undernutrition and its causes, the interviewees’ knowledge and perception of the MAM’Out project, the positive and negative changes induced by the program, and their perception of the use of the mobile phones by women. Participants from the intervention groups were also asked about the types of purchases made with the cash that they received.

One to three days before each interview or focus group, an appointment was made with the participants to ensure their availability. All discussions were conducted in the local language (Gourmanchema for the majority of participants; the discussions were translated into Pulaar in a few cases) and took place in a specifically chosen, quiet location, at home or in the village. Care was taken to ensure the confidentiality of what was reported; only the interviewer(s) and the respondent(s) were present during the interview or the focus group discussion. Each focus group discussion and individual interview lasted between 30 and 45 min. None of the participants were interviewed more than once.

### Data coding and analyses

After participants gave verbal consent, the discussions were recorded, transcribed, and translated into French by a third person skilled in sociology. Due to the high illiteracy rate among women in this rural community, transcripts were not returned to them for comments. Observations made during the interviews were also reported by the interviewers and summarized in written documents. Data obtained from the focus groups and the individual interviews were coded using N-Vivo software (QSR international – version 10) and a written codebook. The coding was performed by combining a deductive approach based on pathways corresponding to the interview guide and the theoretical framework of action of cash transfers, and an inductive approach using an iterative method to integrate emerging pathways (open coding). This enabled us to identify new factors of interest and perceived effects of the cash transfer. Specific attributes of the focus groups and individual interviews were also entered into the N-Vivo software program, such as the socio-economic group that the participants belonged to (very poor versus poor, according to the Household Economy Approach [9]), marital status, and the type of interviewee.

A content data analysis was performed to analyze the results [13]. Summary reports from the two years of data collection were written up based on the identified themes and pathways. The qualitative findings were organized around the main themes described above. Data are presented for all factors of interests, and related quotes are shown in Additional file 2: Tables S1–S3. The differences between these factors according to the attributes are only presented when relevant.

### Quantification of expenses

All mothers in the intervention group were asked three times over the course of the two year project (during the quantitative data collection) how they spent the cash they received and how much they spent. These data were collected at different points during the project: 1/from December 2013 to March 2014, after the first five months of the cash transfer program; 2/from June 2014 to September 2014, during the second round of cash transfers; and 3/from October 2014 to January 2015, corresponding to the end of the second year of the cash transfer program. The interviewers used a questionnaire with seven yes-or-no questions, such as “Did you use the money you received from ACF to buy food for your household?” or “Did you use the money you received from ACF to invest in an income-generating activity?” When participants answered “yes” to a question, they were asked to state the approximate amount spent on this specific activity, using a proportional piling method. Two additional open-ended questions gave participants the chance to report whether they spent the money on other items, and how much. For analysis purposes, the answers to these two additional questions were grouped into one of the following themes: health care, savings, and other.

## Results

### What are the types of purchases made by the beneficiaries of multiannual and seasonal cash transfers?

#### Results from the qualitative data collection

The most commonly reported use of the cash was to purchase food. This can be linked with the words used by the interviewees to describe “malnutrition”. In the Gourmanchema language, this word is defined by various terms. One of the terms used most often by the participants was ″tidjepuari″, meaning “lack of meal”. Even though families were informed at the beginning of the program that the money that was distributed was intended to prevent their youngest children from suffering from acute malnutrition, all participants agreed that most of the money received was spent on food for the child as well as the whole family. This allowed households to improve the quality of the child’s diet and to increase the overall food stores in the house at times when resources were tight, especially from July to September, a period of time corresponding to the hungry period.

The second most commonly reported type of expense was health care; once again, the money was used to support the child’s health, as well as the family’s health. This was indicated by the words used by the interviewees to describe malnutrition in their children, for example: ″li muli″, meaning hemorrhoids, or ″djiérigoji″, meaning worms. Participants also used terms referring to symptoms such as “bloated belly” or “skinny arms” that related to their child’s health status.

Interviewees also indicated that part of the money could have been spent on hygiene products, clothes, and cooking utensils, which can be linked to the term “ti djuanguidi” (in relation to dirtiness), which is also used to describe malnutrition.

Very little investment in income-generating activities was reported in this study. In cases where this was reported (for example, selling bean fritters), it involved the wealthier families included in the study, i.e. the “poor” families. Participants explained that they perceived the monthly cash transfers to have low value in this regard, based on the households’ size and vulnerability. Nevertheless, some beneficiary households reported using part of the money to buy agricultural products and cattle, which can serve as a buffer in times of significant financial constraints.

People also described sharing the money within the household and externally. Generally, once the women reached home with the cash, they gave part of it to their husbands for their private use. A small portion was sometimes shared with the women’s co-wives or mothers-in-law. In some cases, money was shared with individuals who were not part of the household, to maintain inter-household cohesiveness and in keeping with local practices. Participants explained that if a family with sufficient income helps their neighbor, then when the family has resource issues itself, it is likely to receive help in return.

Finally, the focus groups and individual interviews revealed an unexpected type of expenditure. Interviewees reported using some of the money they received to charge the batteries of the mobile phone given to them for cash transfer purposes. They also reported spending some money on phone credits. The amount spent varied between households and depended on how the mobile phone was used.

#### Results from the quantitative data collection

Table 3 summarizes the amount of money spent and types of purchases made with the cash received within the framework of the MAM’Out project. Increasing the household’s food stores and buying food for the child were the two main areas of expenses for the majority of the beneficiary mothers. They estimated that the amount spent each month to increase the household’s food stores was around 5000–6000 FCFA (8–9€) (out of the 10,000 FCFA received), and that around 2800 FCFA (4€) was spent on food for the child. Several of the mothers also reported that they used some of the cash to buy materials for the child’s care (around 1600 FCFA (2.5€)) and for health care for the child and the whole family (around 4500–5000 FCFA (7€)). Money sharing (between 1500 and 3300 FCFA (2–5€)) and savings (2700–4000 FCFA (4–6 €)) also represented a non-negligible part of the 10,000 FCFA that the families received, although this was reported by fewer households. Investment in productive goods and income-generating activities was nearly nonexistent, but when it occurred, the amount spent could be significant.

**Table 3:** Declared amounts and types of purchases made by the intervention group using the cash received – results from the quantitative data collection

		Data collection from December 2013–March 2014		Data collection from June 2014–September 2014		Data collection from October 2014–January 2015	
		Number of individuals who spent money^a^	Mean amount spent (FCFA)	Number of individuals who spent money^b^	Mean amount spent (FCFA)	Number of individuals who spent money^c^	Mean amount spent (FCFA)
Type of expense	Increase food stores for the household	484	4724	390	6391	449	5451
	Buy food for the child	531	2919	315	2787	372	2715
	Invest in another activity (1)	391	3659	163	4693	291	4832
	Buy materials for child care	324	1396	128	1597	288	1844
	Buy non-productive goods	143	1388	24	2392	111	2159
	Invest in another activity (2)	82	2041	31	4064	38	3780
	Share money with husband/parents/neighbor	53	1611	34	3306	26	2973
	Buy productive goods	15	2537	7	5429	14	4071
	Invest in income-generating activities	4	3000	0		1	10,000
Details of other activities (1)	Health care	295	3998	101	5410	228	4824
	Savings	58	2714	48	3258	47	4104
	Other	36		12		16	

The number of individuals presented in the table corresponds to the number of mothers who reported making various types of purchases

^a^Out of 616 respondents

^b^Out of 596 respondents

^c^Out of 592 respondents

### Did the cash transfers induce perceived changes in the daily life of women?

Overall, the heads of households and key members of the community reported that the cash transfers received by the women were well-tolerated.

In terms of changes, both men and women reported that women had an increased participation in decision-making concerning purchases for food and health, as well as in households’ charges, improving communication between husbands and wives. However, beneficiary women reported no change in decision-making regarding traditional health care (i.e. seeking care from traditional healers) for the child: the head of household remained the principal decision-maker in this respect.

Heads of household also expressed being positively surprised by the support they received from their wives, especially regarding purchasing grain for the family. In some cases, this was associated with an encouraging change in the husband’s perception of his wife and of her capacity to make accurate decisions or suggestions. Some participants reported intra-household jealousy from a non-beneficiary woman towards her beneficiary co-wife, as well as misunderstanding between wives and husbands on how the cash should be used.

Most heads of households also reported that their wives managed part of the cash themselves (after giving some to their husbands or other family members) and had increased autonomy. However, traditionally, both men and women have their own “purse” and handle a certain amount of money: men are responsible for buying grain, and women pay for ingredients to make the sauce. However, depending on each household’s rules, women may not be allowed to retain more than a certain amount of money, and may therefore need to give the surplus to their husband, mother-in-law, or parents. In some other Gourmanchema households, the husband controls all of the household’s money and decides how much can be spent on what.

At the community level, social cohesiveness appeared to be reinforced through cash-sharing outside the household and the granting of loans. Participants also reported enhanced communication between women; for example, women who were not included in the study sometimes asked beneficiary women for advice on caring for their children. Some beneficiary women also stated that their relationship with shopkeepers improved due to the cash transfers.

### What are the perceived positive and negative effects of the cash transfer program?

Overall, the MAM’Out program received positive feedback from the beneficiaries and from the community as a whole. At the household level, participants in the focus group discussions reported that the cash transfers increased their purchasing power and reduced the stress and shame that occur when the family is hungry. Families also described having fewer debts or not needing to sell their productive goods and assets. Key village members also reported perceiving positive changes at the community level, particularly the fact that the poorest families did not request financial aid from the richest families. Similarly, members of beneficiary households reported reducing their seasonal migration and paid work on other farmers’ fields in favor of cultivating their own lands.

The MAM’Out research team paid particular attention to any tension that may have arisen due to the program. However, no conflict was reported between beneficiary and non-beneficiary households, both of which perceived the situation as the result of a divine will. Having an intervention and a control group did not seem to create tension or violence between communities, and participants did not describe it as problematic. When control group participants were asked how they felt about not receiving money, they indicated that they hoped to also become beneficiaries.

The participants in the focus group discussions and individual interviews also suggested ways of improving the program. Beneficiary households reported a desire for continuous (not seasonal) cash transfers, and, above all, that the program and the cash transfers would not stop. Participants mentioned that the monetary value of the monthly transfers (around 15€/month) was low, given the households’ size and vulnerability. During the first year, women also expressed dissatisfaction with the distance between their villages and the cash collection points, as well as the amount of time they had to wait before receiving the money. Due to improvements in program management, this was not reported as a weakness during the second year of the program. Misunderstanding of the inclusion criteria for the program, as well as potential corruption within the targeting committee responsible for establishing a list of eligible households, were also reported.

Unexpected effects of the program were also described by some participants, particularly beneficiary women. Some reported not having expected the cash transfers to end, and hoped that the program would continue. Even though the duration of the cash transfer program was made clear to all participants from the beginning of the project, some beneficiary households wondered how they would continue without help. Reduced financial support of beneficiary wives by their husbands was also reported.

Finally, several women, namely mothers and mothers-in-law from both intervention and control villages, reported having heard of non-beneficiary women getting pregnant in order to be part of the cash transfer program the following year, although no further cash transfers are planned. However, no data were available to compare the birth rate in the MAM’Out families to the rest of the population.

## Discussion

The findings regarding expenses from the qualitative data of the study are consistent with the quantitative data. The main uses of the received cash were associated with terms for malnutrition in the local language: money was primarily spent on food and health care for the child and the whole family. Part of the cash was also spent on hygiene and domestic products, and some was shared within the household and with other families. These three types of expenses (food, health care, and products for personal and environmental hygiene) correspond to the three main underlying causes of undernutrition described in the framework of undernutrition reported in the 2008 Lancet series [14]. In this regard, spending mainly in these areas can help prevent undernutrition, and particularly acute malnutrition, in children.

The effects of cash transfer programs are usually assessed using quantifiable indicators. One of the first effects of a cash transfer program is an increase in the household’s income and purchasing power, which in turn has positive effects on food security, through increased spending on food [15, 16]. In Kenya, unconditional cash transfers were associated with greater spending on food, health, and clothing [17]. Cash transfers were also shown to increase access to health care [16, 18]. The qualitative results presented here regarding the purchases made with the distributed cash are thus consistent with results from other studies.

It was hypothesized that transferring cash to women, who do not typically manage household finances in poor rural Burkinabe communities (which are usually led by men), could have effects on their daily lives. One of the major concerns of the MAM’Out research team was the acceptability of this process to male members of the household, who appeared to be satisfied with the women receiving the money.

The cash transfer program lead to several changes in the daily lives of women: they seemed to take on a larger role in decision-making (with regards to health and food purchases), and managed part of the cash that was received, increasing their autonomy. Enhanced communication within the household was also reported, although in some cases, jealousy and misunderstanding arose. At the community level, social cohesiveness appeared to be reinforced through cash-sharing outside the household, and relationships with shopkeepers improved for some families. Increased dignity for the poorest families and a reduction in taking on paid work in other farmers’ fields for some households were also highlighted. Similar results have been reported by other studies. In Malawi, unconditional cash transfers were associated with a drop in income from low-skilled agricultural work [19]. The positive effects of cash transfers on women’s empowerment and personal status have also been described several times. Cash transfers allow recipients to become active members of their households or communities, promoting their self-esteem and empowerment [16]. As mentioned in the 2013 Lancet series, Latin American cash transfer programs were also associated with improvements in women’s control over income and provided opportunities for them to strengthen their social networks [8]. Promotion of women’s empowerment due to cash incentives was also reported in the review by van den Bold et al. [20].

Overall, the MAM’Out program did not create tension between villages, and allowed households to protect their livelihoods and feed family members during the hungry period. However, unexpected perceived effects of the program were also highlighted during the discussions, such as some beneficiaries not expecting the cash transfers to end, and the plans of some women to become pregnant in order to become beneficiaries of the project. This possibility was considered when creating the inclusion criteria and designing the project. In order to reduce this risk of increasing the birth rate, all beneficiaries were chosen on a one-time basis and not continuously. The aim of the program and its duration (two years) were also clearly explained to the women, as a basic ethical consideration, and regular communication about the program occurred throughout the two years. However, these precautions were not sufficient to prevent some women from planning new pregnancies; this point should be investigated further in future research on cash transfers. Pregnancy risks were also analyzed in Fiszbein’s review of conditional cash transfers, which found that these programs had a modest impact on birth rates: some Latin American programs resulted in slightly increased birth rates, while some had no effect [21].

Some other studies have highlighted unexpected effects of such programs, such as misunderstanding of the cash transfer rules, the tensions that these money transfers can create, the perception that less money has been received due to redistribution [22], or even dangerous mystic interpretations that beneficiaries may have of the program [23]. Although misunderstanding of the inclusion criteria and potential corruption were also reported during the interviews, no major security issue or tension was described by the MAM’Out participants. This can be ascribed to the organization of Gourmantché society, in which inter-household solidarity is crucial, unlike in competitive communities. As explained by the participants of the interviews and focus group discussions, an individual who has money now should help his neighbor in need if he wants to be helped in return one day. However, the reported unexpected effects of the transfers may be underestimated. This study only reports the feedback given by participants during the cash transfer program. Thus, they may not have had the necessary perspective to report on potential dependencies that the program could have created, new habits beneficiaries would have to lose, or disillusion after the end of the project.

The concerns and expectations raised by the interviewees bring into question the appropriateness of randomized controlled trials. One of the major aims of this study was to provide evidence for the effectiveness of unconditional, seasonal cash transfers in preventing acute malnutrition in children, so that the Burkinabe government could be petitioned to include this type of strategy in their social protection plan. The qualitative findings are encouraging, and favor longer-term intervention plans. However, solid quantitative results are still needed to provide a more complete understanding of this type of program. The design of this trial will generate reliable evidence.

The significant number of individual interviews and focus group discussions carried out and analyzed over two years lends credence to the results presented here. The variety of participants included in the qualitative data collection provided a broad understanding of different points of view, as well as how they confirmed and/or contradicted each other. From a methodological aspect, the semi-structured questionnaire ensured that accurate information was obtained, while leaving the interviewers and participants free to express their ideas. Desirability bias could be a limitation of the study. However, the significant number of focus groups and individual interviews that were carried out led to saturation: what participants stated in one focus group was generally corroborated by the following one, which suggests that the reported statements were of high quality. Moreover, the topics discussed during the focus groups were confirmed by the individual interviews. Care was also taken to emphasize the potential negative effects of the program, especially during the second year of the program.

The data were collected by trained interviewers each year, although they were not sociologists. This could have resulted in a lower quality of data collection during the first few weeks, particularly during the first year, during which the interviewers did not follow up on many of the participants’ statements. To address this situation, close supervision by a sociologist was implemented during the second year.

The codebook used for analyzing the data was developed by the MAM’Out research team based on a model of how cash transfers could prevent undernutrition in children (Fig. 1) and modified with new themes as they arose. However, it was not peer-reviewed externally. Analyzing the qualitative results in the context of the theoretical framework confirms several pathways. First, cash transfers increased household budgets, and led to an increase in purchasing power, increased spending on food and health, and improved the psychosocial well-being of families (pathways 1 and 3). However, very few investments were made in productive assets. Secondly, the cash transfer program stabilized family budgets, which lead to a decrease in seasonal migration for some families, the retention of productive assets, and repayment of debts and credits (pathways 5, 7 and 8). None of the participants commented on any reduction in income-generating activities at critical points of the year or retaining a portion of the crops that would usually be sold. Therefore, no conclusions can be drawn about these aspects, which should be investigated in future studies. Thirdly, the cash transfer program was associated with a positive change in the role of women, which was also positively perceived by the community. The position of women improved due to the receipt of cash transfers, and women experienced greater control over income and greater decision-making power (pathways 9 and 10). The qualitative data presented in this report thus validate some pathways of the theoretical framework of how cash transfers could prevent undernutrition in children in poor, rural Burkinabe communities. However, these results still need to be confirmed with quantitative indicators.

## Conclusion

Although the cash transfers were unconditional, the money was mainly used to improve the food security and health of children and households, which correspond to two main underlying causes of undernutrition. Spending mainly on these areas can thus help prevent childhood acute malnutrition. The program was also associated with positive perceived changes at the household level, mainly related to gender equality and improvement in women’s status, and promoted social integration of the poorest families at the community level through cash-sharing. Unexpected reported effects associated with this program included some women planning new pregnancies and some individuals not expecting the transfers to end.

## Additional files

Additional file 1: Interview guide used for individual interviews and focus group discussions among beneficiary households. (DOCX 28 kb)

Additional file 2: Table S1. Quotes related to the first question: “What types of purchases were made by the beneficiaries of cash transfers intended to prevent acute malnutrition in children?”. **Table S2.** Quotes related to the second question: “What perceived changes in the daily lives of women were induced by the cash transfers?”. **Table S3.** Quotes related to the third question: “What are the perceived positive and negative effects of the cash transfer program?”. (DOCX 35 kb)

## Abbreviations

ACF: Action Contre la Faim
COREQ: Consolidated criteria for reporting qualitative research
FCFA: West African CFA franc
HEA: Household Economy Analysis
MAM’Out: Moderate Acute Malnutrition Out
WHO: World Health Organization
