# Supplementary material for: Beneficiaries’ perceptions and reported use of unconditional cash transfers intended to prevent acute malnutrition in children in poor rural communities in Burkina Faso: qualitative results from the MAM’Out randomized controlled trial
Source: BMC Public Health. 2017 May 30;17:527. doi: 10.1186/s12889-017-4453-y (PMC5450256; doi:10.1186/s12889-017-4453-y)
Supplement: Supplementary file 1 — Interview guide used for individual interviews and focus group discussions among beneficiary households. (DOCX 28 kb) [file 12889_2017_4453_MOESM1_ESM.docx]

**Guide d’entretien individuel et focus group**

**(A destination des Bénéficiaires Cash)**

**Identification**

Date: ....... /…….. /…….. Nom du village : ______________________

Heure du DEBUT de l’entretien: |___|___| H |___|___|mn

Heure de FIN de l’entretien: |___|___| H |___|___|mn Durée : |___|___|

Nom et prénom de l’enquêté : ___________________________

Fonction/rôle de l’enquêté : ____________________________

Lieu: ____________ ID Animateur : |___|___|

*Bien préciser avant de démarrer l’entretien que tout ce qui sera dit restera confidentiel et qu’aucune réponse n’est ni bonne ni mauvaise. Demander à enregistrer vos échanges. Le but de cet entretien est d’avoir l’avis des participants du projet, pour ensuite pouvoir l’améliorer.*

1. **Perception de la malnutrition**

- Selon vous à quoi renvoie le terme malnutrition? A quoi cela vous fait-il penser ?
- Y a-t-il d’autres appellations que vous utilisez pour parler de malnutrition ?
- Comment se manifeste la malnutrition ?
- Selon vous pourquoi existe-il plus de malnutris garçons que de filles?
- Comment la prévenir selon vous?

1. **Connaissance et contribution du projet**

- Avez-vous été informés du démarrage de la deuxième période de transferts monétaires ?
- Pensez-vous que les bénéficiaires ont été bien prévenues de la reprise ?
- Comment trouvez-vous l’organisation des transferts monétaires ?
- Avez-vous des remarques sur la manière dont cela se passe d’un point de vue pratique (exemple : en termes d’attente, de personnes pour vous aider/servir…) ?
- Que pensez-vous du projet en général (mesures régulières des enfants, questions...) ?
- Avez-vous entendu des commentaires à propos de ce projet (positifs ou négatifs) ? si oui, lesquels ?

1. **Utilisation de l’argent transférés**

- Comment avez-vous utilisé l’argent qui vous a été donné ? A quoi a servi cet argent?
- Pourquoi avoir utilisé cet argent pour ces dépenses ?
- Qui a décidé de l’utilisation de l’argent pour ces différentes dépenses ? (Sonder femmes, époux, belles-mères …)
- Comment avez-vous entretenu vos enfants pendant la période de suspension du cash?
- Pendant cette période, avez-vous été confrontés à des difficultés ? si oui, lesquelles ?

1. **Perception des changements**

- Qu’est-ce que cet argent a apporté de positif dans la vie de votre ménage ? (Sonder Changement au niveau alimentaire, soins, ……)
- Qu’est-ce que cet argent a apporté de négatif dans la vie de votre ménage ? (Sonder conflits internes liés à la gestion, le choix des postes de dépenses)
- Qu’est-ce que cet argent a apporté de positif dans le village ? (Sonder Changement au niveau alimentaire, soins,…)
- Qu’est-ce que cet argent a apporté de négatif dans vos relations avec le voisinage? (Sonder conflits entre ménages, avec les commerçants ….)
- Que pensez-vous des changements que cet argent a apportés dans votre ménage?
- Le cash a-t-il contribué à rendre favorable / améliorer les conditions de vie des femmes? (comparaison des conditions de vie de la bénéficiaire avec sa vie antérieure ou avec la sienne)
- Vous êtes-vous déjà senti mal à l’aise ou en insécurité à cause de ce projet?

1. **Perception de l’utilisation du téléphone portable**

- Quelle appréciation faites-vous de la possession du téléphone portable par les femmes?
- Selon vous, quelles sont les utilisations qu’elles en font?
- Quels sont les avantages liés à son utilisation pour les femmes?
- Selon vous, le téléphone portable pourrait-il être la source de problèmes dans certains ménages ? (exemple : mésentente entre époux et épouse)
- Si oui, pouvez-vous nous décrire les problèmes survenus suite à l’apparition du téléphone portable dans le foyer ?
- A votre avis, quelle est la place que la femme acquiert dans le ménage ou dans le village en possédant le téléphone portable?
- Quel lien faites-vous entre le téléphone portable et le transfert monétaire?
- Que pensez-vous du moyen de transfert utilisé ? Quels sont les avantages ? quels sont les inconvénients ?
- Avez-vous d’autres points que vous souhaiteriez ajouter à la discussion ?

**Merci pour votre collaboration!**

**Individual interviews and focus group discussions guide**

**(For cash beneficiaries)**

**Identification**

Date: ....... /…….. /…….. Village’s name: ______________________

Time of BEGINNING of the discussion: |___|___| H |___|___|min

Time of END of the discussion: |___|___| H |___|___|min Duration: |___|___|

Name of the interviewee: ___________________________

Type of interviewee: ____________________________

Location: ____________ ID Interviewer: |___|___|

*Precise before beginning the interview that all that will being said will stay confidential. No answer is good or wrong. Ask the permission to record the discussions. The aim of the discussion is to have the point of view of the project’s participants, in order to improve it in the future.*

1. **Perception of malnutrition**

- According to you, to what the term « malnutrition » refers to ? What do you think about when you hear this term?
- Do you use other terms to speak about malnutrition?
- How does malnutrition express?
- According to you, why are there more malnourished boys than girls?
- How to prevent malnutrition according to you?

1. **Knowledge and contribution of the project**

- Were you informed about the beginning of the second period of the cash transfers ?
- Do you think that beneficiaries knew about the second period of the cash transfers?
- How do you find the organization of the transfers?
- Do you have any remark on how it occurs from a practical perspective (eg: waiting time, number of people to help/serve you…)?
- What do you think about the project in its whole? (Regular measurement of children, questions…)
- Did you hear any comment concerning this project (positive or negative)? If yes, which ones?

1. **Use of the money that was transfered**

- How did you use the cash that was given to you? What was this money for?
- Why did you use this money for this kind of expenses?
- Who decided about the use of this money for the various expenses that you mentioned? (women, husbands, mothers-in-law…)
- How did you take care of your children during the non-transfer period?
- During this period, did you face any difficulty? If yes, which ones?

1. **Perception of changes**

- What did this money bring from a positive point of view in the daily life of your household? (Changes in food consumption care…)
- What did this money bring from a negative point of view in the daily life of your household? (Tensions regarding management of cash, choice of expenses…)
- What did this money bring from a positive point of view in the daily life of your village? (Changes in food consumption care…)
- What did this money bring from a negative point of view in the relationship with your neighborhood? (Conflicts with other households, shop keepers…)
- What do you think of the changes that the cash brought into your household?
- Did cash contribute to improve women’s status/life? (Comparison of the women’s conditions of life before and after receiving cash)
- Did you already feel insecure or tense because of this project?

1. **Perception of the use of the mobile phone**

- Which appreciation do you have about women having a mobile phone?
- According to you, what uses do they make of the mobile phone?
- Do you think that mobile phone could be the source of tensions in some households? (eg: misunderstanding between spouses)
- If yes, could you describe use the problems that arise because of the mobile phone in the household?
- According to you, which place does a woman have in the household or in the village when having a mobile phone?
- Do you make any link between mobile phones and cash transfers?
- What do you think of the transfer mean that was used? What are the advantages? What are the disadvantages?
- Do you have any other points that you wish to add to the discussion?

**Thank you for your collaboration!**
