# Supplementary material for: Beneficiaries’ perceptions and reported use of unconditional cash transfers intended to prevent acute malnutrition in children in poor rural communities in Burkina Faso: qualitative results from the MAM’Out randomized controlled trial
Source: BMC Public Health. 2017 May 30;17:527. doi: 10.1186/s12889-017-4453-y (PMC5450256; doi:10.1186/s12889-017-4453-y)
Supplement: Supplementary file 2 — Quotes related to the first question: “What types of purchases were made by the beneficiaries of cash transfers intended to prevent acute malnutrition in children?”. Table S2. Quotes related to the second question: “What perceived changes in the daily lives of women were induced by the cash transfers?”. Table S3. Quotes related to the third question: “What are the perceived positive and negative effects of the cash transfer program?”. (DOCX 35 kb) [file 12889_2017_4453_MOESM2_ESM.docx]

Table S1: Quotes related to the first question: “What types of purchases were made by the beneficiaries of cash transfers intended to prevent acute malnutrition in children?”

| Types of purchases/expenses | Quotes |
| --- | --- |
| Food | - “In this period of famine, you use it to buy millet in order to survive the famine and for planting. But I leave a part for the child’s needs: 5000 F. The other 5000 F, it’s for the purchase of millet.” Beneficiary woman - “We can’t only spend it on the child. We lack food; we can’t only feed the child and starve.” Beneficiary woman |
| Health care | - “At the time I took my money, the child I am in charge of was sick and his brother too. I brought them both to consultation. I bought medicines for 3500 F and 4000 F for the younger child and older child, respectively.” Beneficiary woman |
| Hygiene products, clothes, and cooking utensils | - “Often our clothes get dirty and we wear them without having washed them. Now, we have something to buy soap to wash them, for us, for our children and for our husband.” Beneficiary women - “She first bought a cooking pot for the kitchen… During the celebrations, she bought clothes for her child.” Co-wife of a beneficiary woman |
| Investment in income-generating activities | - “The money is insufficient to meet the family’s needs. Therefore we don’t think about investing it in business.” Beneficiary woman - “I told my wife: ‘As we are lucky that the child isn’t ill, we will pay for animals.’ We bought two rams that are there. […] It could be the child’s needs or ours that will lead us to sell them.” Head of a beneficiary household |
| Money sharing | - “I didn’t go over 500 F. This money belongs to the head of household. Even if Action Contre la Faim asks me, it’s my money and I spend it for my needs. The rest, I told her to handle it for the child.” Key village member of the intervention group (and also head of a beneficiary household) - “At home, she gave me 2000 F to buy things for my child.” Co-wife of a beneficiary woman - “When she comes back, she usually gives me 1000 F to buy kola.” Mother-in-law of a beneficiary woman - “Once, she lent 5000 F to another woman who came to tell her about a problem.” Head of a beneficiary household |
| Phone charging | - “We buy phone credits. For me, it’s 500 F per month. I charge the battery three times a month, it costs 300 F. My battery wasn’t good, I bought a new one for 2000 F.” Beneficiary woman |

Table S2: Quotes related to the second question: “What perceived changes were induced in the daily lives of women by the cash transfers?”

| Perceived changes | Quotes |
| --- | --- |
| Acceptance of women as cash transfer recipients | - “I don’t have any problem with my wife. However, the cash transfers improve our love. She hardly bothers me about the little things that the child needs.” Head of a beneficiary household |
| Decision-making | - “We realized that we support our husbands very well. This also makes the women proud to know that they are respected by the family. You know that if you often make gestures, you can’t be ignored during discussions.” Beneficiary woman - “There is a clear improvement. The woman doesn’t wait for her husband’s point of view about her child’s care anymore, except if there isn’t enough money. In that case, she calls me by phone to inform me. That’s what is going on at home. For the household’s maintenance too, there is a clear change.” Head of a beneficiary household - “For Gourmantché people, the husband first has to consult the sand. Then, if there is a sacrifice to be made, he makes it, and if the child isn’t healed, we take him to the health center. So I go through my husband if it is for traditional health care, but if it is the health center I don’t necessarily wait for my husband. I decide to take the child there myself.” Beneficiary woman |
| Men’s perception of their wives | - “When she got the money, she came to tell me: ‘That’s it, there’s no more millet. I suggest that we pay for some millet.’ But I couldn’t open my mouth because for me, it was like a dream that she had such an idea.” Head of beneficiary household - “My husband congratulated me last time because I supported him during the hunger gap.” Beneficiary woman |
| Jealousy / misunderstanding | - “My husband told me to buy an animal for the child and me; I told him that I would rather invest in the child’s food and health. He threatened me by saying that he would report that to Action Contre la Faim, because he said I wasn’t saving any money for the child.” Beneficiary woman - “I would even say that the project didn’t help the child, but helped me. Imagine if I took all the money I should have given to the mother, and used it for other needs. In that case, you know that this small support I give to her co-wife’s children softens the jealousy that her co-wife could express.” Head of a beneficiary household |
| Increased autonomy and management of cash | - “When she came back, she gave me the money and I told her that I travel a lot. I would rather have her keep the money. Since then, it’s my wife who holds the money.” Head of beneficiary household - “Today, the woman supports the man. Instead of bothering us for money, as soon as her money arrives, she can do whatever she wishes without our support. This really pleases us.” Head of beneficiary household |
| Social cohesiveness | - “There is a big change, because a lot of women ask me how I take care of my child and I tell them.” Beneficiary woman - “There is good collaboration, because when money comes, you go to buy something and don’t have to use credit. What shopkeeper doesn’t like such a client!” Beneficiary woman |

Table S3: Quotes related to the third question: “What are the perceived positive and negative effects of the cash transfer program?”

| Perceived positive and negative effects of the program | Quotes |
| --- | --- |
| Decreased feelings of shame | - “It’s a support to our husbands. Our husbands relied on animals breeding to buy millet, but today, because of diseases, the animals aren’t here anymore. With this money, we support our husbands; we cleanse our household of shame.” Beneficiary woman |
| Fewer debts or less selling of productive assets | - “We don’t take out any more loans. Before, you would suffer to get credit and then you suffered to pay it back.” Beneficiary woman - “When I received money, I didn’t sell my goods. But during this break period, I was obliged to sell my chicken to care for him.” Beneficiary woman |
| Positive changes at the community level | - “The money created many changes because the poorest don’t bother the people who manage things anymore. So this helps; everybody takes advantage of it.” Key member of the community - “Some parents, without this money, would be forced to work on other peoples’ farms to earn money or take out credit to cover their children’s needs. Thanks to this money, many of them stay to take care of their fields or carry out their own activities.” Key member of the community |
| No reported conflict | - “All the people who receive money enjoy it, and those who don’t benefit pray to God to be included the next time.” Non-beneficiary woman |
| Ways of improving the program | - “If I am reminded that the distribution will end next year, I’m not happy at all.” Beneficiary woman - “It takes 5000 F to buy millet for all eight of us, and this lasts four to five days maximum.” Beneficiary woman - “For me, it’s to ask you to increase the amount of money. Instead of 10,000 F, 15,000 F would be good. For some children’s illnesses, 10,000 F is not enough.” Beneficiary woman - “Last year it was complicated. But this year no one had to stay in the place, except the ones who had problems with their mobile phones. Last year, sometimes they had to spend two days there.” Beneficiary woman |
| Misunderstanding of inclusion criteria | - “There were some people who said that the village advisor made deals; some others said they enrolled people based on some criteria. So there are people who understand and other who don’t.” Head of beneficiary household |
| Not expecting the transfers to end | - “We became like children today. If a mother goes to the market and brings back biscuits for her child, the child will beg for them next time. So it’s the same for us. If you leave us, we will suffer because we were used to getting money, and that’s it.” Beneficiary woman - “For us, the women, it’s complicated. We don’t have the means; we didn’t do anything to get the money. When I received money, I didn’t wait to take my child to the health center. However, since the distribution stopped, I don’t have anything.” Beneficiary woman |
| Reduced financial support | - “Our husband doesn’t give her any more money now. She already has something and I don’t, so he provides support to me so I can meet my children’s needs.” Co-wife of a beneficiary woman |
| New pregnancy plans | - “What I heard from the neighbor, it’s that she was saying that next year she will also have a new-born in order to benefit from this money.” Beneficiary woman |
